# Effects of Cognitive Training with Virtual Reality in Older Adults: A Systematic Review

**DOI:** 10.3390/brainsci15090910

**Published:** 2025-08-23

**Authors:** Christian Daniel Navarro-Ramos, Joselinn Murataya-Gutiérrez, Christian Oswaldo Acosta-Quiroz, Raquel García-Flores, Sonia Beatriz Echeverría-Castro

**Affiliations:** 1 Technological Institute of Sonora (ITSON), Central Campus, Ciudad Obregon 85000, Sonora, Mexico; christian.navarro11@outlook.com (C.D.N.-R.); joselinn.murataya174179@potros.itson.edu.mx (J.M.-G.); 2 Department of Psychology, Technological Institute of Sonora (ITSON), Ciudad Obregon 85000, Sonora, Mexico; raquel.garcia@itson.edu.mx (R.G.-F.); soniae@itson.edu.mx (S.B.E.-C.)

**Keywords:** virtual reality, cognitive training, older adults, cognitive decline, immersive technology, neurorehabilitation

## Abstract

**Background/Objective:** The use of immersive virtual reality (VR) for cognitive training in older adults has shown promising results in recent years. However, the number of well-designed studies remains limited, and variability in methodologies makes it difficult to draw generalizable conclusions. This systematic review aims to examine the effects of VR-based cognitive training in older adults, describe the technological characteristics of these interventions, identify current gaps in the literature, and suggest future research directions. **Methods:** Following PRISMA guidelines, a search was conducted across major databases (PubMed, PsycINFO, Scopus, ProQuest, ACM, and Web of Science) from 2018 to 2025. The database search identified 156 studies, of which 12 met the inclusion criteria after screening and eligibility assessment. Across these studies, a total of 3202 older adult participants (aged 60 years or older) were included. Interventions varied in duration from 4 to 36 sessions, targeting domains such as memory, executive function, attention, and global cognition. Most interventions were based on cognitive training, with a few employing cognitive stimulation or cognitive rehabilitation approaches. Quality was assessed using the Effective Public Health Practice Project tool. **Results:** Most studies reported positive effects of VR interventions on cognitive domains such as attention, executive functions, and global cognition. Fewer studies showed improvements in memory. The majority used head-mounted displays connected to computers and custom-built software, often without public access. Sample sizes were generally small, and blinding procedures were often unclear. The average methodological quality was moderate. **Conclusions:** Immersive VR has potential as an effective tool for cognitive training in older adults. Future research should include larger randomized controlled trials, long-term follow-up, standardized intervention protocols, and the development of accessible software to enable replication and broader application in clinical and community settings.

## 1. Introduction

Virtual reality (VR) can be defined as inducing directed behavior in an organism through the use of artificial sensory stimulation, while the organism has little or no awareness of the interference [1].

VR offers immersive, multisensory simulation environments that can elicit perceptual, affective, and cognitive responses through the integration of visual, auditory, and proprioceptive stimuli, generating a heightened sense of presence and facilitating active cognitive engagement [2]. Neurobiologically, VR exposure has been associated with enhanced neuroplasticity, including increases in cortical gray matter density and modulated neural activity in beta-band frequencies [3]. In neurorehabilitation, VR has proven valuable for simulating functional tasks with real-time feedback and high repetition, enabling more effective cognitive and motor recovery than traditional methods [3]. Neuroimaging studies indicate that VR-based interventions can induce structural and functional brain changes, such as increased hippocampal volume and enhanced connectivity in networks related to memory and attention [4].

Regarding cognitive functions, meta-analyses have demonstrated that VR interventions can produce moderate improvements in domains such as attention, memory, executive function, and global cognition in older adults with cognitive decline [5,6]. For example, VR training has significantly enhanced memory (effect size g ≈ 0.45), attention/executive function (g ≈ 0.49), and motor outcomes like balance (g ≈ 0.43) [6]. Furthermore, studies indicate that VR interventions are especially relevant for aging populations due to their engaging and motivating nature, which can enhance adherence, an important factor given the challenges older adults may face in accessing or using new technologies [7]. A recent meta-analysis confirmed that immersive VR interventions in older adults with cognitive decline yield significant improvements in memory, attention, and processing speed, albeit with varying quality of evidence [5].

Virtual reality has proven to be a promising tool to help reduce isolation and improve commitment between people. VR components work in tandem to create a sensory illusion that produces a lifelike simulation. These components give users audio, visual, and tactile information through head-mounted displays (HMDs) that provide different degrees of immersion [2].

VR can deliver a complete level of immersion in an interactive space where they can explore without outside interferences. These spaces can mimic scenes where older adults might have limited access or be completely inaccessible. In addition, they can be optimized for the population to adjust to their needs and give them a balance so that there is the correct amount of stimulation [8].

It has been found that the use of augmented reality and virtual reality technologies as a support to improve physical and psychological well-being in older adults has increased, while social well-being is the least studied area. In older ages, developing meaningful relationships with others and maintaining a social network to overcome loneliness is critical, and virtual reality could facilitate social well-being through virtual interactions that promote socialization and improve relationships [9]. VR interventions can improve adherence in older adults by increasing motivation and enjoyment, factors often associated with better long-term cognitive outcomes [10].

On the other hand, a study [11] of the level of acceptance of VR in older adults showed that older adults with no prior VR experience initially had a neutral attitude towards this technology; however, after a first exposure to it, attitudes improved. Therefore, the concern should be about to what extent older adults can learn to use the technology rather than their acceptance of it. Symptoms of cyber-illness (similar to motion sickness) were measured, and no significant complaints were found in the participants. In conclusion, older adults are willing to use VR and may improve their attitudes towards it after using it for the first time.

Although VR interventions for cognitive health have been studied in various populations, there is a notable gap in reviews that focus specifically on older adults at risk of or experiencing cognitive decline. Most existing systematic reviews pool heterogeneous interventions, combining motor rehabilitation, cognitive training, and recreational VR applications without differentiating between their theoretical frameworks or intended outcomes [6,12]. This lack of distinction is critical, as cognitive training, stimulation, and rehabilitation differ in structure, goals, and intensity [13,14]. By narrowing our scope to VR interventions with a primary cognitive focus and clearly classifying them according to these modalities, this review addresses an underexplored yet highly relevant area for aging populations, where tailored cognitive strategies are essential to maintaining independence and quality of life.

Some definitions were proposed to avoid the confusion that has historically existed between cognitive interventions, such as the indistinct and interchangeable use between rehabilitation, training, and stimulation. In first place, cognitive stimulation consists of promoting participation in activities and discussions (usually in a group) that aimed at a general improvement of cognitive and social functioning, without specific objectives [13]. Overall, observational studies have found that increased cognitive stimulation is linked to slower cognitive decline over time [15]. Instead, cognitive rehabilitation is a personalized approach where relevant personal goals are identified and the therapist works with the person and their family to design strategies to address them. The emphasis is on improving performance in daily life, rather than cognitive tests, taking advantage of the individual’s strengths and developing ways to compensate for impairments. Finally, cognitive training consists of the guided practice of standard tasks designed to reflect particular cognitive functions, with the possibility of having different difficulty levels in the tasks to suit each individual’s ability. This can be performed in groups or individually, with pencil and paper, or using a computer [13].

Cognitive training has been demonstrated as being effective in slowing the deterioration in the activities of daily living in old age, extending health and cognitive abilities to reinforce personal effectiveness, preserve autonomy, and improve the social integration of older adults in their own environments. However, there are significant challenges associated with face-to-face cognitive training, so it is necessary to identify quick and effective solutions to slow down age-related cognitive decline. Through the use of technology-based cognitive training programs (computers, smart phones, televisions, tablets, virtual reality, video games, etc.) [16], which can be applied to the elderly population and offer a more flexible approach compared to traditional cognitive training programs. They can provide feedback on cognitive performance in real time and can be adjusted to the user’s ability level, and greater involvement with the activity can be achieved, because the technologies are designed to be fun and to motivate older adults to become more involved with the cognitive training program [12].

Previous systematic reviews have examined the effects of VR on cognitive health in older adults, but many have combined heterogeneous interventions, mixing motor rehabilitation, cognitive training, and recreational use without differentiating between cognitive stimulation, training, and rehabilitation protocols [6,12]. This lack of conceptual clarity makes it difficult to draw precise conclusions about the most effective approaches for specific populations. The present review narrows its focus to interventions primarily grounded in cognitive training with structured and repetitive exercises targeting specific cognitive domains such as memory, executive function, and attention, while also identifying the few studies employing cognitive stimulation or cognitive rehabilitation. This distinction is crucial, as the majority of VR interventions with older adults at risk of cognitive decline emphasize training-based paradigms, whereas stimulation and rehabilitation approaches are less frequently implemented and often follow different theoretical and methodological frameworks [5,17]. By separating these modalities, this review seeks to clarify the specific contributions of VR-based cognitive training in aging populations and to identify gaps for future research.

The objectives of this systematic review (SR) were the following:

- Identify the impact of using VR for intervention.
- Identify the features of VR systems used for cognitive training in older adults.
- Identify gaps in the literature.
- Provide future research directions.

This systematic review aims to help researchers to find the potential benefits and research perspectives regarding the use of VR systems for cognitive training in older adults.

## 2. Materials and Methods

This systematic review has been prospectively registered at PROSPERO (CRD42023408116) and was conducted using the PRISMA (Preferred Reporting Items for Systematic reviews and Meta-Analyses) 2020 methodology [18]. The review was conducted from March 2023 to June 2025. A meta-analysis was not performed because of the considerable heterogeneity among the included studies. Differences were observed in the type of cognitive intervention (training, stimulation, rehabilitation), duration and frequency of sessions, VR hardware and software used, outcome measures, and participant characteristics. This level of variability would have compromised the validity and interpretability of a pooled effect size. Consequently, we opted for a narrative synthesis, which allowed for a more detailed interpretation of the findings and facilitated the identification of patterns, methodological trends, and contextual factors influencing the effectiveness of VR-based cognitive interventions.

### 2.1. Inclusion and Exclusion Criteria

Studies were selected according to the following inclusion and exclusion criteria:

Inclusion criteria (IC)

IC-1—The paper has one of the following terms in the title, abstract, or keywords:

“Virtual Reality” or equivalent expressions.

“Older Adults” or equivalent expressions.

“Cognitive Training” or equivalent expressions.

IC-2—Only peer-reviewed research articles reporting empirical data were included. Conference abstracts, theses, book chapters, editorials, and other non–peer-reviewed sources were excluded.

IC-3—The paper is written in English or Spanish.

IC-4—The paper is a pre-experimental, quasi-experimental, experimental, or randomized clinical trial or a pilot study.

Exclusion Criteria (EC)

EC-1—The paper is unavailable.

EC-2—The paper does not consider immersive virtual reality.

EC-3—The paper does not evaluate cognition before and after intervention.

EC-4—The study is theoretical (e.g., proposes a framework).

### 2.2. Search Strategy

The first stage of the search strategy consisted of data retrieval from databases by entering the search string in the following databases: PubMed, Web of Science, PsycINFO, ProQuest, ACM, and Scopus. To guarantee a more comprehensive search, the search started with a string representing the three main aspects of this review, “virtual reality’’ AND “older adults” AND “cognitive training”. Then, synonyms and related words were added. Thus, the following search string was defined: (“virtual reality” OR VR) AND (“older adults” OR elderly OR “older people”) AND (“cognitive training” OR “cognitive stimulation” OR “cognitive rehabilitation”). The established year limits were from 2018 to 2025; all the related research was recovered. The decision to focus on studies published from 2018 onward was based on the technological shift brought by stand-alone headsets, which increased accessibility and reduced the costs of VR-based cognitive interventions [19]. The retrieved papers were imported to Mendeley using the BibTex format. This permitted us to erase the duplicated papers and adjust and export the final papers to a spreadsheet.

### 2.3. Study Selection

For every retrieved paper, the title, abstract, and keywords were assessed with the eligibility criteria. This phase involved the reading of each abstract of the retrieved papers in a blinded standardized manner by two researchers, independently. In cases where there was no consensus, a third reviewer made the final decision, these articles were retained for more careful analysis in the phase of full-text analysis.

We included randomized clinical trials using fully or partially immersive virtual reality as a cognitive training tool in older adults (≥60 years), with pre- and post-intervention cognitive measures. Studies that used augmented reality, did not have a control group, or were aimed at other pathologies not related to cognitive impairment were excluded.

### 2.4. Data Collection Process

For each included study, we extracted the following pieces of data: authors, year of publication, country, study design, sample characteristics (age, sex, cognitive status), intervention type (cognitive training, stimulation, or rehabilitation), intervention duration and frequency, cognitive domains assessed, outcome measures, the main results, and VR hardware and software used (namely an online platform, an application, or a game). For studies that used applications that were built solely for the study, we reported if the software was made available for public use or not. Data on methodological quality were also recorded according to the Effective Public Health Practice Project (EPHPP) tool.

Data extraction was independently conducted by two reviewers (Author 1 and Author 2) using an Excel spreadsheet. Disagreements were resolved through discussion, and when necessary, a third reviewer (Author 3) acted as arbiter.

All extracted data and quality assessment files were stored securely in a password-protected cloud folder accessible only to the review team. A backup was also maintained on an encrypted external hard drive to ensure data integrity and prevent loss.

### 2.5. Data Analysis

Due to the methodological and outcome heterogeneity across studies (differences in VR systems, cognitive domains assessed, intervention duration, and measurement tools), a quantitative meta-analysis was not feasible. Instead, we employed a narrative synthesis approach to summarize and compare the effects reported in each study. Data extracted included study design, participant characteristics, type of cognitive intervention (training, stimulation, or rehabilitation), VR hardware and software used, session frequency and duration, and cognitive outcomes assessed. Findings were categorized according to the targeted cognitive domains (e.g., memory, attention, executive functions) and intervention type. This process was conducted independently by two reviewers, and any discrepancies were resolved through discussion to ensure consistency and reliability in the synthesis.

In this review, the term effects refer specifically to the cognitive outcomes measured in the included studies. These outcomes encompass changes in specific cognitive domains such as memory, executive functions, attention, and processing speed, as assessed through validated neuropsychological tests. Where applicable, we also considered secondary effects reported by the studies, including changes in mood, engagement, or motivation. This operational definition ensured consistency in the extraction, synthesis, and interpretation of results across studies.

### 2.6. Quality Assessment

For the methodological quality assessment, we selected the EPHPP Quality Assessment Tool [20]. This instrument was chosen because it is designed to evaluate a wide range of quantitative study designs, including randomized controlled trials, quasi-experimental studies, and observational research. Unlike the PEDro scale, which is primarily focused on physiotherapy interventions and emphasizes aspects relevant to physical rehabilitation, the EPHPP tool allows for a broader assessment that captures the multidisciplinary nature of VR-based cognitive interventions. These interventions often integrate cognitive, psychosocial, and motor components, making the EPHPP tool’s domains (e.g., selection bias, confounders, data collection methods) more appropriate for the scope of the included studies. The EPHPP tool was selected for its applicability to a variety of study designs, a key advantage in fields where randomized controlled trials are limited [20]. In this system, the score of 1 means a Strong score rating, a 2 means a Moderate rating score, and a 3 means a Weak rating score. The quality rating was given according to eight components: Selection Bias, Study Design, Cofounders, Blinding, Data Collection Methods, Withdrawals and Drop-outs, Intervention Integrity, and Analysis.

## 3. Results

### 3.1. Selected Studies

A total of 156 studies were identified. After removing duplicates and applying the inclusion and exclusion criteria, 12 studies were included in the final review, of which eight studies were published between 2018 and 2023 and four were published between 2024 and 2025. These correspond to randomized clinical trials and systematic reviews with meta-analyses of high methodological quality.

The included studies consistently report improvements in executive functions, sustained attention, and, to a lesser degree, memory. For example, Ref. [21] evaluated a motor-cognitive intervention with immersive virtual reality in 293 older adults, finding significant improvements in global cognition and frailty. Refs. [6,22] reported significant positive effects on attention and executive inhibition. Ref. [5], based on 30 studies, identified improvements in global cognition and quality of life, as well as functional benefits. Effect sizes reported for attention and executive function were moderate, while effects on memory were more variable. All interventions used virtual reality headsets and interactive software designed for specific cognitive tasks.

Although the term cognitive stimulation was initially used as an umbrella term, the majority of the included studies fall under the category of *cognitive training*, characterized by structured, repetitive tasks aimed at improving specific cognitive functions. Additionally, one study employed a cognitive stimulation approach and another focused on cognitive rehabilitation in the context of physical and cognitive decline. Despite their conceptual differences, these modalities share the goal of enhancing or preserving cognitive functioning in older adults. Their inclusion in this review is justified by the common use of virtual reality as a delivery method and the need to better understand the diverse ways in which VR is being integrated into cognitive interventions for aging populations.

**Figure 1:**
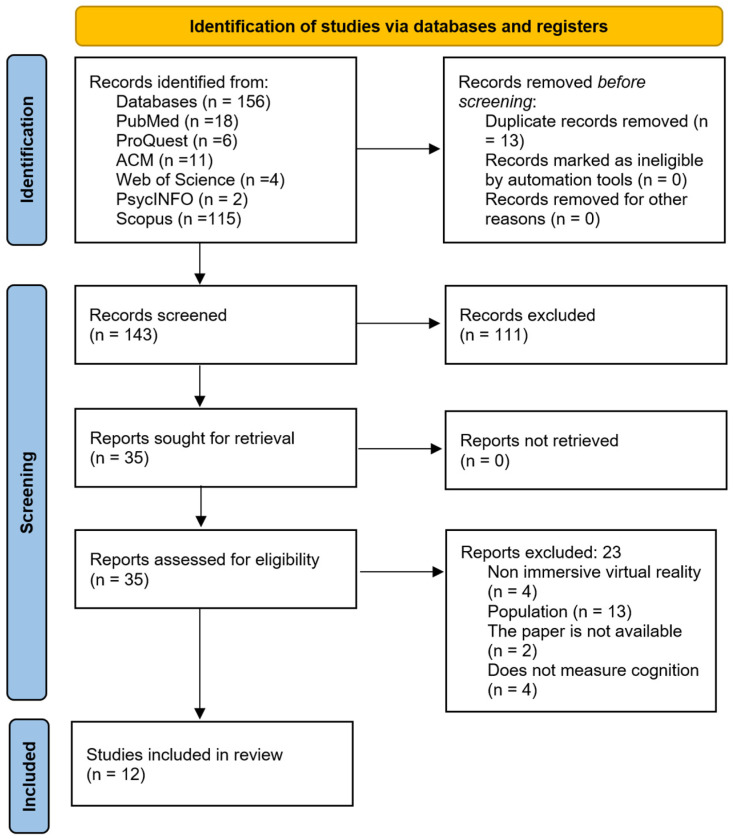
PRISMA flowchart of study selection.

Figure 1 presents a flow diagram with a report of the outcomes obtained in each phase. In Table 1, we display a synthesis of the key characteristics extracted from the articles that were analyzed and selected and the variables collected and introduced in the data collection process to address the objectives initially stated.

### 3.2. Quality Assessment Results

The scoring of the 12 papers ultimately included in this SR revealed an average global rating of 2.33, meaning that most of the studies had a Moderate global rating (Table 2).

### 3.3. Study Characteristics

#### 3.3.1. Sample

All the studies included in this review worked with older adults aged 60 years or older. A total of 3202 older adults participated across the twelve full-text papers included, giving an average of 39.5 participants per study. Most studies included both an intervention group and a comparison group. One study used a single-group design with pre–post assessment.

#### 3.3.2. Intervention

Two studies had a one-time intervention session, while the remaining nine implemented intervention programs that ranged from 2 weeks to 12 weeks, with session lengths varying from 10 min to 100 min. The frequency of sessions varied from twice a week to daily. All interventions were delivered in-person in clinical, research, or community-based settings.

#### 3.3.3. Measures

The measures used in the studies were appropriately selected for both screening and pre–post intervention evaluation. The most common screening tool was the Mini-Mental State Examination (MMSE). For outcome assessment, most authors used multiple instruments tailored to their study’s objectives, covering domains such as attention, executive functions, visuospatial ability, and memory.

#### 3.3.4. Were People Trained to Use the VR Systems Before the Intervention?

Six studies did not train their participants in the use of VR prior to the intervention. The remaining five used structured strategies, including familiarization sessions, demonstrations with assistance, or formal protocols based on instructional design models (such as ADDIE). These strategies aimed to increase participants’ confidence and reduce technological anxiety.

#### 3.3.5. VR Devices Used for Interventions

All studies specified the hardware used for the intervention. The most commonly used headset was the HTC Vive, followed by Oculus Rift and Oculus Quest 2. One study also incorporated hand-tracking technology to increase interaction and immersion within the virtual environment. All interventions used immersive VR headsets, although some were tethered to PCs, limiting mobility.

#### 3.3.6. Software Used for Interventions

All studies used custom-developed VR software tailored to their study’s cognitive goals. One study also integrated the commercially available software *Job Simulator* by Owlchemy Labs as part of its stimulation environment. None of the studies provided access to their developed software, limiting replication. All the studies developed their own software for their respective intervention.

#### 3.3.7. Outcomes

Across the twelve included studies, the effects observed primarily concerned cognitive domains such as memory, executive functions, attention, and processing speed. Several studies also reported secondary benefits, including improved mood, increased engagement, and higher intervention adherence. These effects were measured using a range of validated instruments, and their magnitude and statistical significance varied according to the type of cognitive intervention applied and the study design. According to eight of the reviewed papers, immersive VR had a positive impact on cognitive performance in older adults, particularly in attention and executive functioning. The remaining three reported no significant cognitive changes following the intervention. None of the studies reported any negative cognitive or psychological effects related to VR use.

## 4. Discussion

### 4.1. The Impact of VR-Based Interventions

In interpreting the findings of this review, it is important to recall that the term effects refer to changes in targeted cognitive domains such as memory, executive functions, attention, and processing speed, as measured through standardized neuropsychological assessments. While the magnitude of these effects varied across studies, a consistent trend emerged for moderate improvements in at least one cognitive domain in most interventions. Additionally, several studies documented secondary effects, including enhanced motivation, higher adherence, and improved mood, which, although not the primary focus of this review, provide valuable insight into the broader impact of VR-based cognitive training in older adults.

The present systematic review synthesized evidence on the effects of VR interventions targeting cognitive functions in older adults at risk of or experiencing cognitive decline. Across the included studies, most reported significant improvements in at least one cognitive domain, most frequently executive function, attention, and memory, following VR-based cognitive interventions. Even in studies where cognitive changes did not reach statistical significance, VR was shown to be at least as effective as traditional cognitive training programs, supporting its potential as a viable alternative. Compared to previous reviews that included heterogeneous interventions, our focused approach on cognitive training allows for more precise identification of effective strategies [30].

The results from this review show that most of the selected studies reported a positive impact on cognitive domains, especially executive functions and sustained attention [6,7,17,21,23,24,25]. Even when cognitive improvements were not statistically significant in some domains (like memory), the studies still demonstrated that VR was at least as effective as traditional methods of cognitive training [6,26].

One of the key developments in the most recent studies is the inclusion of participants with cognitive frailty [21], showing that immersive VR interventions can be adapted to more vulnerable populations without sacrificing feasibility or safety. Moreover, meta-analyses confirmed moderate to large effect sizes in cognition, particularly for attention and executive functioning [31].

Although the majority of the included studies implemented cognitive training interventions [7,12,21,25,28,29,31], there was also one study focused on cognitive stimulation [Rute] and one on cognitive rehabilitation [17,26]. This distribution has important implications for interpreting the results. Cognitive training, with its structured and repetitive practice targeting specific cognitive domains, tends to yield more consistent improvements in measurable outcomes such as executive function and attention. In contrast, cognitive stimulation typically emphasizes broader engagement through varied and less structured activities, which may lead to more diffuse cognitive benefits. The single rehabilitation study targeted functional recovery in individuals with specific impairments, making its results less directly comparable to the rest. This heterogeneity in intervention type may partly explain the differences in effect sizes and statistical significance across the included studies and highlights the need for clearer standardization in future research designs.

Several recent systematic reviews and meta-analyses have examined the use of VR in older adults, often reporting moderate improvements in cognitive domains such as attention, memory, and executive function [5,6]. Our findings are consistent with these trends, particularly regarding the benefits of immersive VR training on executive function and processing speed. However, unlike previous reviews that have pooled heterogeneous interventions, including motor rehabilitation, recreational VR, and mixed cognitive–motor programs, our study differentiates between cognitive training, stimulation, and rehabilitation protocols. This distinction is important because previous evidence suggests that the intensity, specificity, and theoretical underpinnings of each modality can influence cognitive outcomes in older populations [12,17]. By focusing primarily on cognitive training interventions while still acknowledging the presence of stimulation and rehabilitation approaches, our review provides a clearer understanding of the mechanisms and expected effects of VR in populations at risk of cognitive decline. These findings also align with reports that intervention tailoring and targeted cognitive domain training may enhance neuroplasticity and functional outcomes in aging populations [8,28].

Not all studies included in this review reported significant cognitive improvements following VR interventions, a finding that mirrors earlier work in which certain protocols, particularly those with shorter durations, lower training intensity, or limited domain specificity, failed to produce measurable gains [22,27,29]. These mixed results highlight the importance of optimizing dosage, task complexity, and ecological validity when designing VR-based cognitive interventions for older adults. Moreover, methodological limitations such as small sample sizes, lack of active control groups, and inconsistent follow-up assessments may partially account for the variability in outcomes across studies [22,24]. Addressing these design challenges will be crucial to establishing more robust evidence and ensuring that VR interventions can deliver consistent and clinically meaningful benefits in cognitive health among aging populations.

If we review specific methodological aspects in each of the selected studies, we find that in Cho et al., [21], this randomized controlled trial (RCT) examined VR-based dual-task training in participants with cognitive frailty. The authors reported significant improvements in executive function and attention compared to controls, consistent with meta-analytic findings on VR’s capacity to enhance divided attention through enriched multisensory engagement. However, the short follow-up period limited conclusions regarding long-term maintenance.

The meta-analysis included in Yang et al., [22] reported moderate-to-large effect sizes for attention and executive function in older adults with cognitive impairment. Although this synthesis strengthens the evidence base, its inclusion of heterogeneous protocols (from simple VR tasks to complex training) means that the pooled effects may overestimate the impact of more structured interventions.

In Yu et al., [6], the systematic review and meta-analysis focused exclusively on patients with mild cognitive impairment (MCI), finding significant benefits for global cognition and processing speed. However, the included trials were small and varied widely in intensity, with some protocols providing fewer than five sessions, meaning they were potentially insufficient for neuroplastic changes.

In Li et al., [5], an RCT-based meta-analysis concluded that VR cognitive training yielded significant gains in memory and executive functioning. The emphasis on structured, repetitive tasks aligns with the cognitive training paradigm, supporting the premise that targeted exercises are more effective than general cognitive stimulation for this population.

In Yun et al., [23], a feasibility study explored the usability of fully immersive VR training for patients with MCI and mild dementia. Participants tolerated the intervention well, and qualitative data indicated high motivation. Nevertheless, the absence of a control group and the small sample size (n < 20) restricted the robustness of the findings.

In Liao et al., [24], an RCT combining VR-based physical and cognitive training demonstrated improvements in executive function and dual-task gait. This aligns with dual-task theory, suggesting that motor–cognitive integration can amplify benefits. However, the reliance on a tethered VR system may reduce ecological validity and scalability.

In Park, [25], a single-study RCT focused on hippocampal activation via spatial navigation VR tasks in MCI patients. Imaging results supported neurobiological engagement of memory-related networks, but behavioral cognitive outcomes were modest—a reminder that neural activation does not always translate to functional improvements.

In Lima et al., [26], a rehabilitation-focused trial targeted balance disorders in older adults, finding concurrent improvements in visuospatial attention. Although not primarily designed as a cognitive intervention, the results support the cross-domain benefits of VR when physical and cognitive demands are integrated.

In Zhu et al., [7], a pilot pre–post study using immersive VR cognitive stimulation in MCI and mild dementia patients reported significant improvements in perceived stress and mood alongside cognitive gains. The lack of a comparison group and small sample size limit causal inference but suggest psychosocial benefits as an important secondary outcome.

The RCT in Makmee & Wongupparaj, [28] found significant improvements in executive functions among healthy older adults, supporting VR’s applicability beyond clinical populations. However, the absence of long-term follow-up raises questions about durability.

In Thapa et al., [29], a trial tested a multi-domain VR intervention in MCI patients, finding significant improvements in global cognition. However, the outcome measures varied widely across domains, making it difficult to pinpoint the mechanisms driving change.

In Ferrer et al., [17], a pilot cognitive rehabilitation protocol for MCI showed usability and preliminary cognitive benefits. This is one of the few interventions framed as rehabilitation rather than training, suggesting that rehabilitation-based VR may be viable but requires larger-scale testing.

### 4.2. Interpretation of Findings

Our findings indicate that VR-based cognitive training with structured, repetitive exercises targeting specific cognitive domains was the predominant intervention type in the reviewed literature. Only a small number of studies employed cognitive stimulation (general, non-targeted engagement of cognitive processes) or cognitive rehabilitation (individualized, clinically guided therapy). Training-based protocols tend to yield more consistent, measurable outcomes than less structured interventions [12], a distinction that is critical when evaluating the efficacy of VR interventions for cognitive improvement.

Across training-based interventions, improvements in executive function and attention were consistent, often with medium effect sizes [6], while memory improvements were more variable, potentially due to differences in intervention intensity, duration, and cognitive baseline status.

### 4.3. Implications for Research and Practice

VR interventions are particularly valuable for older adults due to their immersive nature, engaging multisensory experience, and capacity to simulate daily-life tasks with real-time feedback—factors that enhance motivation and adherence [7]. However, scalability challenges persist, including reliance on tethered hardware and in-person delivery, as well as the limited availability of standardized software tools [5].

By clarifying the conceptual differences between cognitive stimulation, training, and rehabilitation, this review provides a framework that can guide future interventions toward greater methodological consistency and better replication of findings.

### 4.4. Rationale for Narrative Synthesis

A meta-analysis was not conducted due to significant heterogeneity across studies in terms of intervention modality, session structure, outcome measures, and participant profiles, rendering pooled effect sizes unreliable [3]. A narrative synthesis therefore allowed for a more nuanced interpretation of the evidence.

### 4.5. VR Systems Used for Cognitive Training in Older Adults

All the studies continued to use VR headsets connected to a computer, limiting mobility within the virtual environment. Only one study used a wireless HMD [25], but even then it was tethered to a PC during the sessions. None of the reviewed interventions used stand-alone VR headsets (e.g., Meta Quest), which could simplify implementation in real-world contexts.

In addition, all interventions were delivered in-person, typically at research facilities or clinics. While this approach ensures safety and technical support, it also restricts the scalability of interventions by limiting their use community- or home-based settings, which are crucial for broader adoption.

### 4.6. Sample Sizes and the Ability to Replicate Interventions

None of the reviewed studies had a sufficiently large sample to generalize their findings. Most had fewer than 50 participants per group, and even the more recent randomized trials did not overcome this limitation [21]. This is understandable due to the nature of the target population, but it limits the strength of the conclusions.

Another persistent issue is replicability. For every study that developed its own software for cognitive training using VR, none of these tools were made publicly available. This lack of standardization hinders the accumulation of comparable evidence and limits the external validity of findings. This prevents other researchers from testing, validating, or improving the interventions. If we aim to advance this field, the software used in clinical trials should be shared either through commercial licensing or open access to enable replication and broader evaluation across contexts.

### 4.7. Methodological Quality

The methodological quality of the included studies, assessed using the EPHPP tool, was generally moderate. Most studies were designed as randomized controlled trials and reported using validated cognitive outcome measures. However, common limitations were observed across the literature, including small sample sizes, limited reporting on blinding procedures, and a lack of publicly available software for replication. These factors, while not undermining the potential of VR-based cognitive interventions, highlight the need for more robust and transparent methodologies in future research.

### 4.8. Limitations of the Review

Key limitations of this work include the small number of eligible studies and the generally small sample sizes, which may limit statistical power. Methodological variability, the lack of long-term follow-ups, and potential publication bias further constrain generalizability. Additionally, the 2018–2025 search window focused on contemporary VR technologies but may have excluded earlier foundational studies.

### 4.9. Contribution and Added Value

This review is among the first to clearly distinguish between different cognitive intervention frameworks such as stimulation, training, and rehabilitation within VR-based studies involving older adults. This conceptual clarity helps to identify research gaps and informs the future design of VR-based cognitive interventions. Future research should explore standardized VR cognitive protocols to enhance replicability across diverse clinical and community settings [32].

## 5. Conclusions

This review highlights that virtual-reality–based cognitive training is not only feasible for older adults at risk of cognitive decline but also capable of producing meaningful improvements in key domains such as memory, attention, and executive function. Beyond its cognitive benefits, VR offers a uniquely immersive and engaging experience that can enhance motivation and adherence, two essential factors in the success of any intervention for aging populations.

What sets this review apart is its clear conceptual distinction between cognitive training, stimulation, and rehabilitation, an approach often overlooked in prior reviews. By focusing on training-based interventions while still identifying examples of the other two modalities, this work provides a sharper lens for interpreting outcomes and guiding future research.

The evidence points to a promising but underdeveloped field. Most interventions are still experimental, involve small and homogeneous samples, and use bespoke software that remains inaccessible to other researchers. Addressing these limitations through larger trials, standardized protocols, and the sharing of tools will be critical for moving from promising pilot studies to scalable, evidence-based solutions.

Ultimately, VR-based cognitive training has the potential to transform how we support cognitive health in older adults. By combining technological innovation with rigorous scientific methods, future research can unlock its full capacity to promote healthy aging and maintain cognitive vitality.

## Figures and Tables

**Table 1:** Key study characteristics.

Study	Sample	Intervention	Measures	Previous Training	VR Device Used	Software Used (Game or App)	Outcome
[23]	11 older adults aged 65 or older with mild cognitive impairment or mild dementia.	The participants experienced 4 harvest games and 3 cooking games one time each.	MMSE, CDR, and GDS, were assessed.	There was no previous training before the intervention with the VR device.	The HTC Vive and a hand tracking module were used for the participants to control in-game interactions.	The authors developed a virtual game of harvesting and cooking in a rural environment representing Korea. The software is not available for public use.	There were no statistically significant differences between pre and post MMSE, CDS or GDS.
[24]	34 older adults aged 65 or older (18 in physical exercise group and 16 in cognitive program group).	Each group received 36 sessions of 60 min each in a span of 3 months. The physical exercise group used a Kinect system and programs for resistance exercise, aerobic exercise, and functional tasks in the forms of window cleaning, goldfish scooping, and other tasks relevant to daily activities. The cognitive training group used a proprietary software program and “*Job Simulator*” for the intervention.	TMT parts A and B and SCWT.	There was no previous training before the intervention for any group.	HTC Vive for cognitive program, and Kinect for physical exercise program.	For the immersive VR intervention, they used a proprietary software developed for the study (not available for public use) and a commercial game called “*Job Simulator*” from Owlchemy Labs.	Both groups showed significant improvements in executive function, single-task gait performance, and motor dual-task gait, but only the VR group showed improvements in cognitive dual-task gait performance and the dual-task costs of cadence after training.
[25]	56 older adults aged 60 or older with amnestic mild cognitive impairment, which were divided into an experimental group (*n* = 28) and a control group (*n* = 28).	8 weeks of VR spatial cognitive training, 45 min sessions, 3 times a week.	WAIS-BDT and SLVT.	The authors did not provide training for using the devices before the intervention.	It is not clear which VR HMD device was used.	They developed their own software for the study; it is not available to the public.	Results suggest that the VR intervention might be beneficial in enhancing the spatial cognition and episodic memory of older adults with mild cognitive impairment.
[26]	37 older adults aged between 60 and 79 years. Participants were divided into a control group (*n* = 17) and an experimental group (*n* = 20).	Each group member participated in 16 individual 50 min morning sessions twice a week. The experimental group used 4 virtual reality games that covered different motor and cognitive demands such as attention, concentration, memory, jumping, crouching, etc. The control group performed balance training through an exercise with a protocol to stimulate the tripod of systems responsible for maintaining postural control: the visual, somatosensory, and vestibular systems.	Static balance was assessed using the Clinical Test of Sensory Interaction and Balance (CTSIB), mobility using the Timed Up and Go Test (TUG), and reach using the Functional Reach Test (FRT).	Before the intervention, participants went through a familiarization session with the HMD.	Oculus Rift	Four games were selected to use in the intervention: “*BoxVR*”, “*Baskhead*”, “*InCell*”, and “*Thrills and Chills Roller Coasters*”. All of these games are available for public use, either by purchase or free access.	There were no significant differences in the outcomes of both groups, with both types of intervention being shown to be effective when analyzed in as intra-group manner.
[7]	31 older adults aged 60 years or older (18 with mild cognitive impairment and 13 with mild dementia)	Participants were instructed to train 3 times per week over a 5-week period and to complete 3 training sessions on each training day, with each session lasting 20–30 min. The program involves training in multiple cognitive domains, such as memory, attention, executive function, and calculation ability.	Auditory Verbal Learning Test (AVLT), the Shape Trail Test (STT), the Symbol Digit Modalities Test (SDMT), GDS, and The Chinese Perceived Stress Scale (PSS).	Participants were taught how to wear the head-mounted display and use its controls to interact with the virtual supermarket during 3 separate 10 to 15-min sessions. Multiple exercises were used to help the participants adapt to the virtual environment.	HTC Vive Pro Eye	They developed a personalized immersive VR cognitive management program for older adults that was divided into 3 modules: an operation learning module, a cognitive evaluation module, and a cognitive intervention module. The software is not available for public use.	The mild dementia group had significantly greater improvement in general cognitive function than the mild cognitive impairment group. This difference may be due to the personalized difficulty setting of the software. Adjusting the appropriate task difficulty according to the basic cognitive level of patients with mild dementia has a positive impact on improving their cognitive function.
[27]	20 older adults (mean age was 79 years, SD 7.8) with clinical dementia ratings ranging from very mild dementia to moderate dementia.	The VR intervention was administered twice per week over a period of 3 months. The participants viewed and interacted with the VR content for approximately 10 to 12 min each time.	Participants were assessed with the Cognitive Abilities Screening Instrument (CASI), MMSE, the global status by Clinical Dementia Rating (CDR), and the depressive symptoms by Center for Epidemiological Studies Depression (CESD).	Participants did not receive previous training to prepare them for the intervention.	HTC Vive Pro	The authors created a software program based on a historical type of residence that was commonly found throughout Taiwan in 1960–1980. The participants could use the controllers to turn on a radio to play music and to open a photo album to browse the photographs with a narrating voice. They could also use the controllers to hold rice to feed chickens, which was a tradition in older villages. The software is not available for public use.	There were no significant changes in cognition, global status, and caregiver burden after the intervention, but depressive symptoms improved significantly afterwards. Immersive VR reminiscence may improve mood and preserve cognitive function in elderly patients with dementia during the period of intervention.
[28]	60 community-dwelling older adults were recruited and equally and randomly assigned to the control (*n* = 30) and experimental groups (*n* = 30). The mean age of all participants was 65.87 ± 4.18 years.	The experimental group received the VR training in the form of two 60 min sessions on a weekly basis for 4 weeks. The control group did not receive an intervention.	The Go/noGo test was used to index the inhibitory control as a central component of executive function. Forward and backward digit span tests were applied to tax abilities to update and manipulate information in working memory. Berg’s Card Sorting Test (BCST) was used to measure the executive function features switching task.	They used the analysis, design, development, implementation, and evaluation (ADDIE) model as an important phase for improving the VR learning environment and performance of the participants in the study.	Oculus Quest	The authors developed a VR software to fulfill six psychological processes (sensory memory, encoding, planning, movement control, active manipulation, and adaptation). The software is not available for public use.	The intervention significantly improved the executive functions of older adults in the experimental group. Specifically, the major enhancements were observed for inhibition, as shown by the response time, updating, as represented by the memory span, and the response time and shifting abilities, as indexed by the percentage of correct responses, respectively.
[29]	68 older adults (mean age was 72.5 years) who were randomly allocated to a control (*n* = 34) and a VR-intervention (*n* = 34) group.	24 sessions of VR-based cognitive training for eight weeks. Three sessions were held per week and each VR training session lasted for 100 min, which also included instruction regarding VR training and eye stretching exercises in between VR training.	MMSE-Dementia Screening, TMT parts A and B and symbol digit substitution test (SDST).	They did not mention any kind of training for VR usage prior to the intervention.	Oculus Quest	The VR software training program consists of four types of games: Juice making; Crow Shooting; Fireworks (count the number of fireworks); and Love house (memorize objects in a house). The software was developed for the study and is not available for public use.	The intervention group exhibited a significantly improved executive function and brain function in a resting state. Additionally, gait speed and mobility were also significantly improved after the follow-up. The VR-based training program improved cognitive and physical function in patients with mild cognitive impairment relative to controls.
[21]	*N* = 293, adults aged ≥65 years with cognitive fragility	Motor-cognitive VR (dual-task), 16 sessions (60 min), 2x/week, 8 weeks	MMSE, Trail Making Test A/B, Stroop, verbal memory	Not reported	Head-mounted display (brand not specified)	Developed by the authors; cognitive and physical tasks	Significant improvement in global cognition (*p* = 0.03), trend in executive function (*p* = 0.07), reduction in frailty (*p* = 0.03)
[5]	*N* = 1365 older adults with MCI (60+ years)	Immersive and semi-immersive VR interventions aimed at older adults with MCI. Variable duration and frequency between studies included in the meta-analysis.	MoCA, MMSE, Digit Span (F/B), IADL	Variable according to study	Different types according to the study	Interactive simulations, memory, attention, and problem-solving tasks	SMD significant in attention (0.61), working memory (0.89), and functionality (0.22)
[22]	*N* = 722 older adults aged ≥65 years with MCI	Interventions with cognitive, physical, or combined VR (dual-task), focused on activities of daily living and specific cognitive tasks. Duration between 3 and 12 weeks.	Memory, attention, executive function (varied between studies)	Variable	Semi-immersive and immersive	Simulations of daily life, navigation, spatial memory	Positive effects on memory, attention, and executive functions (small to moderate effects)
[6]	*N* = 525 older adults aged ≥60 years with MCI	Fully immersive VR applied to orientation, memory, and attention tasks. Frequency 2 to 3 times a week for 4 to 10 weeks depending on the study.	MMSE; attentional and executive tests	Variable	Head-mounted display (HMD)	Cognitive training tasks: memory, orientation, divided attention	Significant improvements in attention, executive functions, and global cognition but not in memory

MMSE: Mini-Mental State Examination; CDR: Clinical Dementia Rating; GDS: Geriatric Depression Scale; TMT: Trail Making Test; SCWT: Stroop Color and Word Test; WAIS-BDT: Weschler Adult Intelligence Scale-Revised Block Design Test; SVLT: Seoul Verbal Learning Test; IADL: Instrumental Activities of Daily Living.

**Table 2:** Studies scoring from the EPHPP.

Study	Selection Bias	Study Design	Cofounders	Blinding	Data Collection Methods	Withdrawals and Drop-Outs	Global Rating
A	Weak	Weak	Weak	Weak	Strong	Not applicable	Weak
B	Moderate	Weak	Weak	Moderate	Strong	Moderate	Moderate
C	Strong	Strong	Moderate	Weak	Moderate	Strong	Moderate
D	Moderate	Strong	Moderate	Weak	Weak	Moderate	Weak
E	Weak	Weak	Moderate	Weak	Moderate	Moderate	Weak
F	Moderate	Weak	Weak	Weak	Weak	Moderate	Weak
G	Weak	Weak	Weak	Moderate	Strong	Weak	Weak
H	Strong	Strong	Strong	Weak	Moderate	Strong	Moderate
I	Moderate	Strong	Moderate	Weak	Strong	Strong	Moderate
J	Moderate	Strong	Moderate	Not applicable	Strong	Not applicable	Moderate
K	Moderate	Strong	Moderate	Not applicable	Strong	Not applicable	Moderate
L	Moderate	Strong	Moderate	Not applicable	Strong	Moderate	Moderate

## Data Availability

Not applicable.
